# The Potential of Novel Chitosan-Based Scaffolds in Pelvic Organ Prolapse (POP) Treatment through Tissue Engineering

**DOI:** 10.3390/molecules25184280

**Published:** 2020-09-18

**Authors:** Julia Radwan-Pragłowska, Klaudia Stangel-Wójcikiewicz, Marek Piątkowski, Łukasz Janus, Dalibor Matýsek, Marta Kot, Marcin Majka, Dalia Amrom

**Affiliations:** 1 Department of Biotechnology and Physical Chemistry, Faculty of Chemical Engineering and Technology, Cracow University of Technology, Warszawska 24 Street, 31-155 Cracow, Poland; marek.piatkowski@pk.edu.pl (M.P.); lukasz.janus@doktorant.pk.edu.pl (Ł.J.); 2 Gynecology and Oncology Department, Jagiellonian University Collegium Medicum, Kopernika 23, 31-501 Kraków, Poland; klaudia.stangel-wojcikiewicz@uj.edu.pl (K.S.-W.); daliamrom@gmail.com (D.A.); 3 Faculty of Mining and Geology, Technical University of Ostrava, 708 00 Ostrava, Czech Republic; dalibor.matysek@vsb.cz; 4 Transplantology Department, Jagiellonian University Collegium Medicum, Wielicka 265, 30-663 Kraków, Poland; marta.kot@uj.edu.pl (M.K.); mmajka@cm-uj.krakow.pl (M.M.)

**Keywords:** chitosan, biomaterials, pelvic organ prolapse

## Abstract

The growing number of female reproductive system disorders creates a need for novel treatment methods. Tissue engineering brings hope for patients, which enables damaged tissue reconstruction. For this purpose, epithelial cells are cultured on three-dimensional scaffolds. One of the most promising materials is chitosan, which is known for its biocompatibility and biodegradability. The aim of the following study was to verify the potential of chitosan-based biomaterials for pelvic organ prolapse regeneration. The scaffolds were obtained under microwave-assisted conditions in crosslinking reactions, using dicarboxylic acids and aminoacid as crosslinkers, including l-glutamic acid, adipic acid, malonic acid, and levulinic acid. The products were characterized over their physicochemical and biological properties. FT–IR analysis confirmed formation of amide bonds. The scaffolds had a highly porous structure, which was confirmed by SEM analysis. Their porosity was above 90%. The biomaterials had excellent swelling abilities and very good antioxidant properties. The cytotoxicity study was performed on vaginal epithelial VK2/E6E7 and human colon cancer HCT116 cell lines. The results showed that after certain modifications, the proposed scaffolds could be used in pelvic organ prolapse (POP) treatment.

## 1. Introduction

Disorders of the female reproductive system affect women not only during their perimenopausal years, but also during their childbearing age. Depending on the procedure and treatment, there might be physical changes to the urogenital organs, both internally and externally. These changes can have a severe impact on the genitourinary tract, including sexual dysfunction. Currently available therapies, such as laser treatments or administration of hyaluronic acid or serum, are neither sustainable nor supported by clinical research. Therefore, there is a great need in designing a new material that is not only safe but that can also provide structural corrections, while maintaining optimal vulvo-vaginal aesthetics and allowing for a pain-free intercourse [1,2,3,4].

Many recent studies strived to create an ideal tissue scaffold that can be clinically utilized in various tissues. Developing an optimal scaffold biomaterial for surgical procedures would be a breakthrough solution in regenerative medicine. Within the field of urogynecology, the production of three-dimensional scaffolds and their potential application in treating damaged female organs remains the goal of many clinicians [5].

Over the past 25 years, stress urinary incontinence and pelvic organ prolapse were treated by implanting a poly(propylene) mesh that provided structural support [6]. However, there is a lot of controversy regarding this procedure, since serious complications were reported in about 15% of women who underwent mesh implantation. It appears that the safety of poly(propylene) mesh in urogynecologic procedures was not studied adequately in the past [6,7,8]. Thus, while there are limited reported complications from the application of the mesh in the abdomen, site-specific adverse reactions occur when the mesh was implanted in the pelvic floor. These adverse effects could be explained by the presence of a different microbial flora, mechanical properties, and blood supply in the paravaginal organs [7,8].

In 2017, a newly designed estradiol-releasing electrospun poly-l-lactic acid mesh was developed as a potential solution. The mesh closely resembled the healthy tissues in the pelvic floor organs and released 17-β estradiol, which enhanced blood flow to the tissues, stimulated collagen synthesis while inhibiting matrix metalloproteinases and inflammatory responses [9]. The authors of the study observed increased metabolic activity as well as collagen content in adipose-derived mesenchymal stem cells that were cultured on estradiol meshes, relative to control meshes. There was also a significant increase in the vascularity of tissue that was grown on estradiol-releasing poly-l-lactic acid mesh. This was explained by a dose-dependent ability of estradiol to stimulate endometrial endothelial cells to release vascular endothelial growth factor [6]. As such, this newly designed mesh could not only be used in urogynecology but also in other tissue-engineering procedures that focus on stimulating angiogenesis [10,11,12,13,14,15].

Chitosan is a natural polymer composed of randomly arranged subunits made of β (1→4)-d-glucosamine and *N*-acetyl-d-glucosamine. It is obtained from chitin, the building block of the exoskeleton of arthropods and the cell walls of fungi, via the *N*-deacetylation reaction [16,17]. In this process, the amide group on C2 *N*-acetyl-d-glucosamine is cleaved, which generates a free amino group. It can be carried out using sodium hydroxide or via enzymatic routes [16,17,18,19].

Most of the characteristics of this polymer, including its biodegradability, its biological functions, and the physical properties of its solutions, determine its potential clinical applications. These characteristics depend primarily on its deacetylation degree (DD) and on its molecular weight [16,17,18,19].

Due to its versatile properties, chitosan is utilized not only in tissue engineering but also in dietary supplements, hydrogel wound dressings, hemostatic agents, cosmetics, antimicrobial mouthwash products and environmental protection processes, such as wastewater treatment. Numerous studies demonstrated that chitosan is biodegradable and biocompatible. In addition, it is considered relatively safe, since it is biofunctional and lacks antigenic and toxic properties. Furthermore, the presence of hydroxyl and amine functional groups allows the polymer to participate in numerous chemical and enzymatic modifications. In turn, water-soluble chitosan derivatives can be isolated, and the polymer can be used to immobilize ions and release therapeutic compounds [16,17,18,19].

In the biomedical field, the chemical properties of chitosan render it a safe component of scaffolds and dressings, due its resemblance to natural extracellular matrix components, namely glycosaminoglycans (GAGs). For example, the positive free amino groups on its surface interact with the negatively charged surface of the intestinal or respiratory epithelium. In turn, intercellular connections in the epithelium are weakened, which increases the absorption of administered compounds. These features can provide future application in mucosal vaccines, as they would extend the antigenic exposure and facilitate contact with M cells, which would augment the efficacy of vaccination [20,21,22,23,24].

In this article, an attempt was made to verify the potential of chitosan-based scaffolds in urogynecology. The results showed that the proposed chitosan scaffolds have the potential to treat pelvic organ prolapse. However, they require certain modifications to eliminate the phenomenon of local pH decrease, due to the release of acidic crosslinkers.

## 2. Results and Discussion

### 2.1. FT–IR Analysis of the Potential Scaffolds

**Figure 1:**
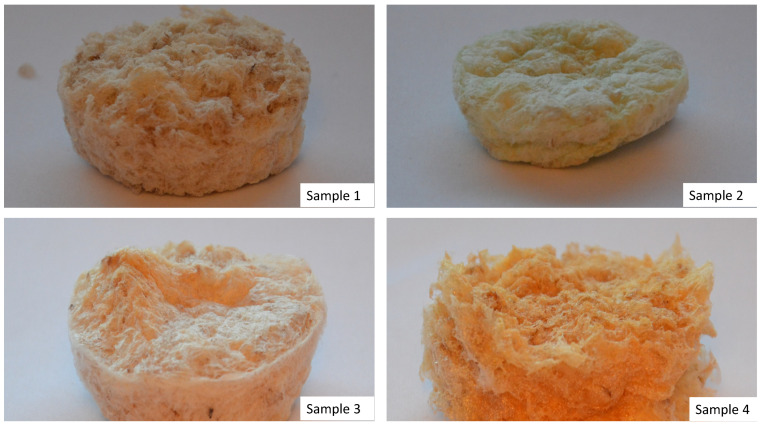
Prepared samples (sample 1—chitosan crosslinked with adipic and malonic acid; sample 2—chitosan crosslinked with adipic and l-glutamic acid; sample 3—chitosan crosslinked with adipic, l-glutamic and malonic acid; sample 4—chitosan crosslinked with adipic and levulinic).

**Figure 2:**
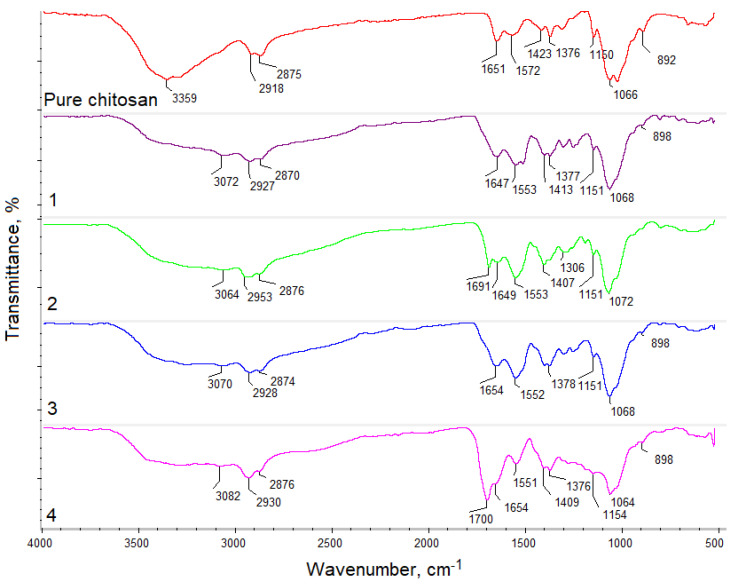
FT–IR analysis of the pure chitosan and obtained samples.

There were four different samples subjected to the study, which varied by their chemical composition (Figure 1). Figure 2 presents FT–IR spectra of the pure chitosan and crosslinked aerogels.

The spectrum of pure chitosan exhibited bands that are typical to this polymer. Free hydroxyl groups could be noticed at 3359 cm^−1^, and free amino groups were present in deacetylated mers at 1572 cm^−1^ and 1150 cm^−1^. Bands typical for amide bonds present in *N*-acylated mers appeared at 1651 cm^−1^. Bands typical for glycosidic bonds between chitosan units were visible at 1066 cm^−1^ and bands characteristic of pyranose ring at 892 cm^−1^ were also visible. Additionally, bands typical for aliphatic groups were noticeable at 2918 cm^−1^ and 2875 cm^−1^, as well as at 1423 cm^−1^ and 1376 cm^−1^, respectively. FT–IR spectrum of the samples 1–4 showed some changes that confirmed the crosslinking reaction. First, it could be noticed that after crosslinking under microwave-assisted conditions, new carboxyl groups appeared, probably due to the slight surface degradation (sample 1—3072 cm^−1^, sample 2—3064 cm^−1^, 3—3070 cm^−1^, and 4—3082 cm^−1^). Intense bands at 1647 cm^−1^ (sample 1) and at 1654 cm^−1^ (sample 3) proved formation of amide bonds between free amino groups and carboxylic groups from the applied acids. On the other hand, one could observe that in the case of samples 2 and 4, the crosslinking process occurred between free hydroxyl and free carboxyl groups. This could be confirmed by the presence of bands at 1691 cm^−1^ (sample 2) and 1700 cm^−1^ (sample 4), corresponding to the newly formed ester bonds. More importantly, FT–IR spectra of all aerogels still showed bands coming from amino groups of various intensity, which suggested that biomaterials are capable of interaction with the cell membrane components of cultured cells. Additionally, bands typical for aliphatic groups, glycosidic bonds, as well as pyranose rings were still present, which confirmed that chitosan did not undergo decomposition during the reaction [25,26,27,28,29].

### 2.2. Porosity and Density Study

**Figure 3:**
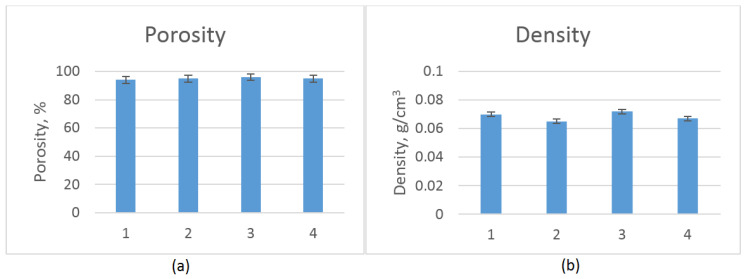
(**a**) Porosity of the prepared scaffolds; and (**b**) density of the prepared scaffolds.

Biomaterials dedicated to tissue engineering must be characterized by low density and high porosity, which should be at least 90% [23]. Multiple pores with interconnecting channels provide nutrients and oxygen delivery and enable CO_2_ and metabolite removal. Such porosity is also needed for the new tissue formation in three dimensions as well as angiogenesis and neovascularization processes. Figure 3 shows that all samples met the aforementioned requirement of excellent porosity. It could be noticed that this parameter was correlated with density and was below 0.06 g/cm^3^, which is typical for aerogels.

### 2.3. Swelling Capability Study

**Figure 4:**
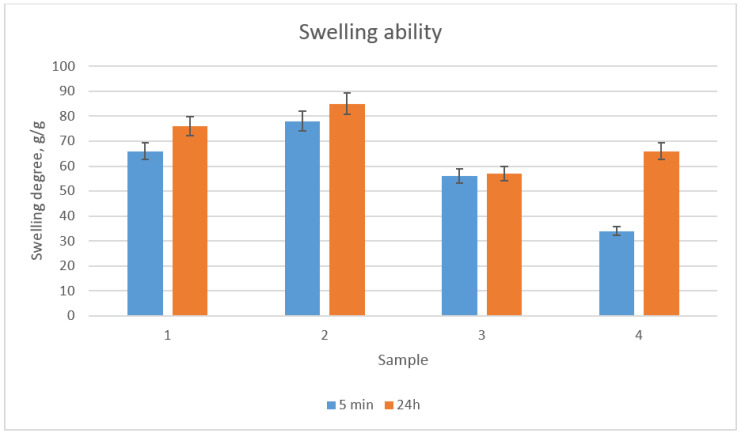
Swelling ability of the prepared scaffolds after 5 min and 24 h in distilled water.

Scaffolds dedicated to regenerative medicine applications besides high porosity should exhibit good swelling properties, in order to provide an appropriate environment for cells growth [24,25,26,27,28,29]. The constant access to culture medium is crucial for cell proliferation. Figure 4 shows the results of the swelling ability study. It could be noticed that all samples had excellent sorption properties. The highest swelling degree could be observed for sample 2, which could be assigned to the presence of the highest number of free hydrophilic groups (hydroxyl, amino, carboxyl) as well as very high porosity [29]. Therefore, it could be assumed that the prepared scaffolds could mimic the natural environment of the cells.

### 2.4. Morphology Study

**Figure 5:**
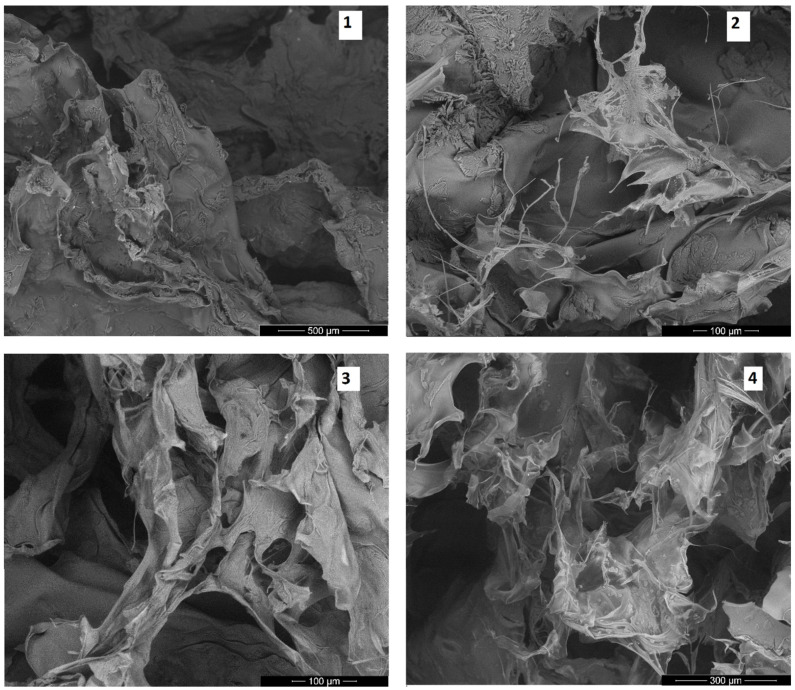
SEM microphotographs of the prepared scaffolds (sample 1—chitosan crosslinked with adipic and malonic acid; sample 2—chitosan crosslinked with adipic and l-glutamic acid; sample 3—chitosan crosslinked with adipic, l-glutamic and malonic acid; sample 4—chitosan crosslinked with adipic and levulinic).

An important feature of the scaffolds is their morphology, which affects cells attachment, migration and the neovascularization process. Figure 5 shows the SEM microphotographs of the obtained biomaterials. It could be noticed that they were all highly porous. Moreover, the pores were open and interconnected. What is important, all pores had diameter above 50 µm, which would enable cell migration inside the three-dimensional structure, as well as new blood vessel formation. In the case of samples 1 and 2, surface microfolding was observed, which could positively affect cell adhesion. The pore edges of samples 1 and 3 were quite smooth as compared to samples 2 and 4, whose pore edges were a little bit sharp. The morphology provided in Figure 5 suggests that the scaffolds could provide good conditions for cell attachment, proliferation, and migration, followed by the formation of the new tissue in three-dimensions, along with the neovascularization process. Very high porosity suggests that the scaffolds would be able to imitate the extracellular matrix at a high level [24,29].

**Figure 6:**
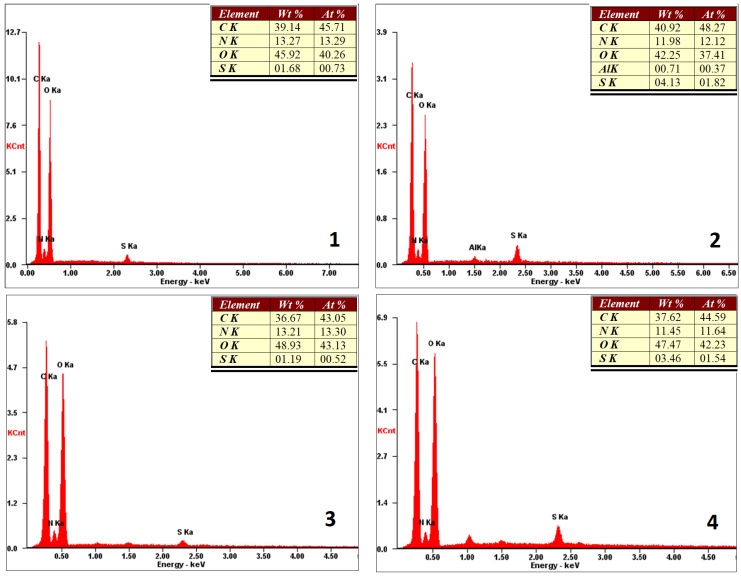
XRF microanalysis of the prepared scaffold surfaces (sample 1—chitosan crosslinked with adipic and malonic acid; sample 2—chitosan crosslinked with adipic and l-glutamic acid; sample 3—chitosan crosslinked with adipic, l-glutamic and malonic acid; sample 4—chitosan crosslinked with adipic and levulinic).

The first stage of the three-dimensional cell culture is cell attachment to the scaffold and surface proliferation [29]. Therefore, it is crucial to know its composition. FT–IR analysis provided information about the chemical bonds and functional groups present in the biomaterials. XRF analysis (Figure 6) presents the elemental composition of the external part of the scaffolds. It was observed that it consisted of carbon, oxygen, and nitrogen. Additionally, some trace amounts of sulfur (all samples) and aluminum (sample 3) could be observed. However, their quantity was negligible. Therefore, it could be assumed that the surface of the scaffolds contained NH_2_ groups, which might interact with the negatively charged cell membranes of various cells, resulting in their adhesion.

### 2.5. Antioxidant Activity Study

**Figure 7:**
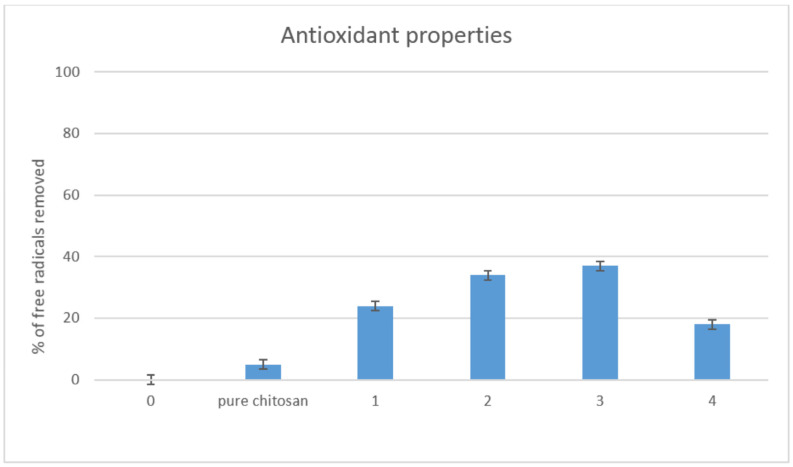
Antioxidant properties study.

Materials for biomedical applications should be characterized by advanced properties, so as to enhance cell growth and proliferation. Scaffolds with three-dimensional structure could protect cells from the external environment and minimize temperature or humidity fluctuations. However, there are other factors that could lead to cells apoptosis. Free radicals constitute a danger to cells. Chitosan is known for its antioxidant properties [25,26]. Nevertheless, polymers dissolved in acidic medium show the best ability for removing free radicals. Figure 7 presents the results of studying the properties of antioxidants. It could be noticed that undissolved chitosan exhibited a low ability of free radicals scavenging, contrary to the obtained scaffolds. Figure 6 shows that all evaluated samples had good antioxidant properties. The best results were obtained in the case of sample 3. The ability of DPPH radicals’ removal could be assigned to the presence of free amino groups, as well as glucopyranose rings. Such results suggest that the scaffolds would be able to protect cultured cells from reactive oxygen species (ROS), which could be generated inside the human body.

### 2.6. Cytotoxicity Study

To determine the cytotoxicity of the scaffolds, proliferation studies were carried out on the VK2/E6E7 epithelial vaginal cell line, as well as HCT116 colorectal carcinoma cell line. Surprisingly, none of the cultured lines (HCT116, VK2/E6E7) proliferated in the expected manner, even those coated with fibronectin.

**Figure 8:**
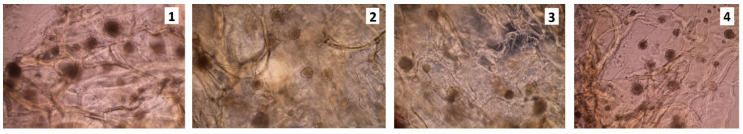
HCT116 cell culture on the scaffolds sterilized with ethylene oxide, rinsed in PBS with the addition of antibiotics; 7th day of the cell culture (sample 1—chitosan crosslinked with adipic and malonic acid; sample 2—chitosan crosslinked with adipic and l-glutamic acid; sample 3—chitosan crosslinked with adipic, l-glutamic and malonic acid; sample 4—chitosan crosslinked with adipic and levulinic)

**Figure 9:**
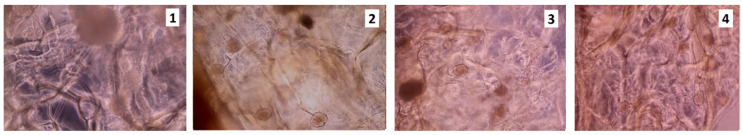
HCT116 cell culture on the unsterilized scaffolds, rinsed in PBS; 5th day of the cell culture (sample 1—chitosan crosslinked with adipic and malonic acid; sample 2—chitosan crosslinked with adipic and l-glutamic acid; sample 3—chitosan crosslinked with adipic, l-glutamic and malonic acid; sample 4—chitosan crosslinked with adipic and levulinic).

**Figure 10:**
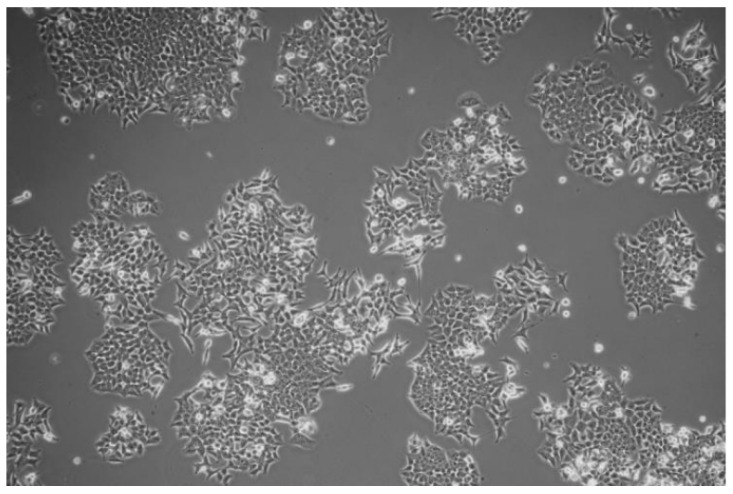
Standard cell culture of the HCT116 cells.

The HCT116 cancer cells adhered to each other rather than to the scaffold and created numerous cell clusters (Figure 8 and Figure 9). The number of living cells compared to the control (Figure 10), was significantly lower.

**Figure 11:**
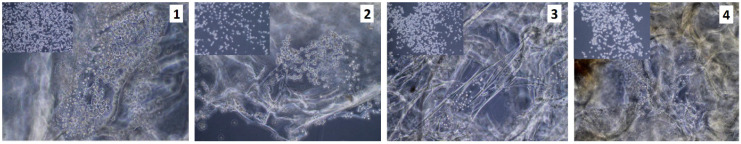
Uncoated with fibronectin, unsterilized scaffolds, rinsed in antibiotics; 3rd of the cell (sample 1—chitosan crosslinked with adipic and malonic acid; sample 2—chitosan crosslinked with adipic and l-glutamic acid; sample 3—chitosan crosslinked with adipic, l-glutamic and malonic acid; sample 4—chitosan crosslinked with adipic and levulinic) culture.

**Figure 12:**
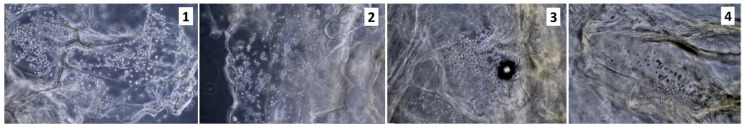
Uncoated with fibronectin, scaffolds sterilized with ethylene oxide, rinsed in antibiotics; 3rd of the cell culture (sample 1—chitosan crosslinked with adipic and malonic acid; sample 2—chitosan crosslinked with adipic and l-glutamic acid; sample 3—chitosan crosslinked with adipic, l-glutamic and malonic acid; sample 4—chitosan crosslinked with adipic and levulinic).

**Figure 13:**
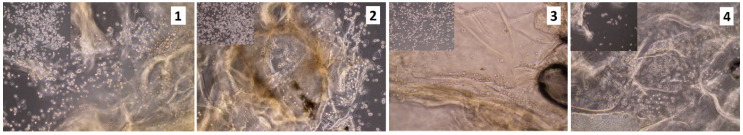
Unsterilized scaffolds coated with fibronectin (100 ng/1 mL), rinsed in antibiotics; 3rd of the cell culture (sample 1—chitosan crosslinked with adipic and malonic acid; sample 2—chitosan crosslinked with adipic and l-glutamic acid; sample 3—chitosan crosslinked with adipic, l-glutamic and malonic acid; sample 4—chitosan crosslinked with adipic and levulinic).

**Figure 14:**
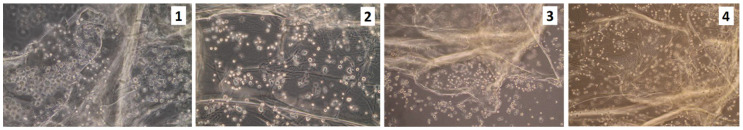
Unsterilized scaffolds coated with fibronectin (100 ng/1 mL), rinsed in the high volume of PBS containing antibiotics; 3rd of the cell culture. (sample 1—chitosan crosslinked with adipic and malonic acid; sample 2—chitosan crosslinked with adipic and l-glutamic acid; sample 3—chitosan crosslinked with adipic, l-glutamic and malonic acid; sample 4—chitosan crosslinked with adipic and levulinic).

**Figure 15:**
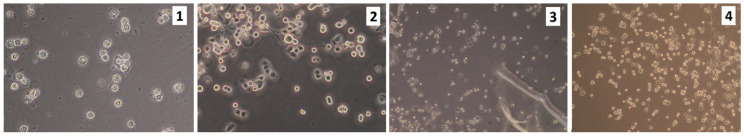
VK2/E6E7 cells on the edges of the scaffolds (sample 1—chitosan crosslinked with adipic and malonic acid; sample 2—chitosan crosslinked with adipic and l-glutamic acid; sample 3—chitosan crosslinked with adipic, l-glutamic and malonic acid; sample 4—chitosan crosslinked with adipic and levulinic).

**Figure 16:**
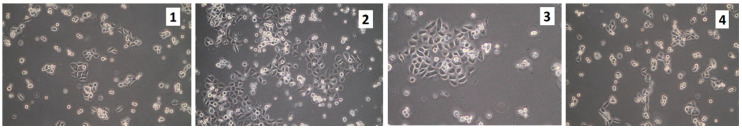
Cells seeded on the scaffolds and then transferred after few hours to a new hole containing fresh culture medium (sample 1—chitosan crosslinked with adipic and malonic acid; sample 2—chitosan crosslinked with adipic and l-glutamic acid; sample 3—chitosan crosslinked with adipic, l-glutamic and malonic acid; sample 4—chitosan crosslinked with adipic and levulinic).

The VK2/E6E7 cells did not flatten and did not proliferate. Such results suggest that certain ingredients in the media negatively affected the cell’s growth (Figure 11, Figure 12, Figure 13, Figure 14, Figure 15 and Figure 16). The cells also did not grow at the bottom of the well, where there was no scaffold present (small photo set in large photographs).

**Figure 17:**
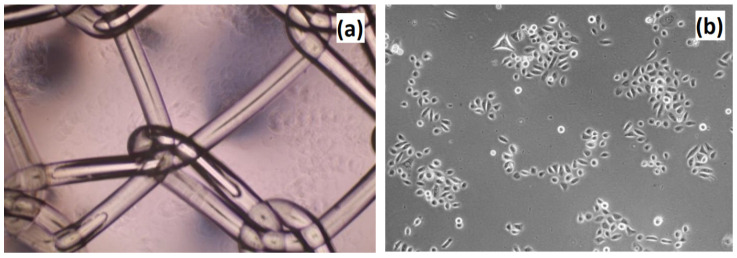
Cell culture of VK2/E6E7 cells on the poly(propylene) mesh—commercially applied biomaterial for pelvic organ prolapse (POP) treatment. (**a**) Poly(propylene) mesh placed in the hole; and (**b**) VK2/E6E7 cells adhered to the bottom of the multi-hole plate.

**Figure 18:**
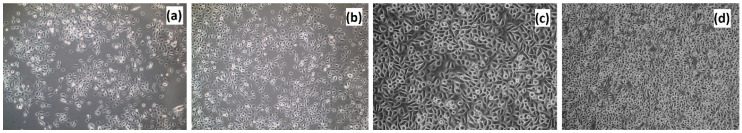
Control culture of VK2/E6E7 cells: (**a**) 1st passage, 5th day of the cell culture; (**b**) 1st passage, 7th day of the cell culture; (**c**) 3rd passage, 7th day of the cell culture; and (**d**) 3rd passage, 7th day of the cell culture.

After plating the VK2/E6E7 cells on scaffolds, some cells were harvested after approximately 8 h and were then plated in new wells with fresh medium. The cells grew in size, flattened, and started to proliferate (Figure 16). However, the cultures were in a much worse condition, relative to the control. Thus, it was likely that the components of the scaffold dissolved in the medium and negatively affected the cellular growth. Rinsing the scaffolds in a large volume of PBS for a few days did not help. Figure 17 presents the VK2/E6E7 cells cultured in the presence of commercially used poly(propylene) mesh. It could be noticed that the cells did not growth on the mesh. However, the cells grew in an appropriate manner on the bottom of the culture flask, which meant that the mesh was not cytotoxic to them. Figure 18 presents VK2/E6E7 cultured under standard conditions. When comparing Figure 11, Figure 12, Figure 13, Figure 14, Figure 15 and Figure 16 to Figure 18, it could be noticed that the amount of viable, flattened cells in the case of the cultures carried out on the scaffolds was significantly lower.

VK2/E6E7 cells were more resistant to external factors than primary cells. Therefore, the cytotoxic effect of the scaffolds was unexpected, especially as numerous studies confirmed the biocompatibility of the chitosan-based materials [27,28,29,30,31]. It could be observed that the cytotoxicity of the scaffolds was not correlated with their sterility. Additionally, it could be concluded that the application of ethylene oxide as a sterilizing agent did not significantly affect their chemical structure and did not affect the cell culture. Taking into consideration the cell culture studies, the synthesis parameters, and the FT–IR and XRF analyses, it could be deduced that the crosslinking process did not occur with the 100% efficiency, leading to the presence of free acids inside them. In all probability, free acids that were not chemically bonded to the chitosan interacted electrostatically with the polymeric functional groups. Thus, they were very hard to wash out using distilled water and PBS. It could be assumed that these were slowly released during the cell culture, leading to a culture medium with a significant pH value decrease.

It is not very likely, that the crosslinked chitosan was cytotoxic by itself, since the previous study on the L929 cell line and MSC primary cells showed that it was not only non-toxic, but also had a positive impact on cell proliferation [25,26,27,28]. However, previously described chitosan scaffolds were prepared by using only one or two aminoacids at the time. In all probability, the combination of dicarboxylic acid, which are much more acidic than acid containing one carboxyl and one amino group, negatively affect the crosslinking process and in the future, the composition of crosslinkers should be better adjusted and the ready products purification method should be refined. Otherwise, their cytotoxic effect is comparable to the currently used polypropylene materials and their in vivo application might be associated with some serious side-effects, such as the emergence of inflammatory states, As compared to alternative solutions like biomaterials prepared from collagen or estradiol [8,9].

The scaffolds did not decompose after being stored in the medium in an incubator (37 °C), for a month, which meant that the materials were stable in the cell culture medium and could be potentially applied in a long-term cell culture, after certain modifications. 

## 3. Materials and Methods

### 3.1. Materials

Glucose, NaOH, HCl, FeSO_4_, NaCl, tetrahydrofuran, methanol, ethanol, acetone, acetonitrile, formic acid, and tartaric acid were purchased from POCH, Gliwice, Poland. l-aspartic acid, Tris-HCl, sodium acetate, diethylene glycol (DEG), *N*,*N*′-Dicyclohexylcarbodiimide (DCC), 4-Dimethylaminopyridine, pyrogallol, rhodamine b, XTT assay, mouse fibroblasts (L929 cell line), Dulbecco’s Modified Eagle Medium DMEM, streptomycin/penicillin (10%), trypsin, and PBS were purchased from Sigma-Aldrich, Poznań, Poland. Molecular weight cut-off 500–1000 Da dialyzing membranes and filter membranes (0.22 μm) were purchased from Bionovo, Zielona Góra, Poland. All reagents were of analytical grade purity.

### 3.2. Methods

#### 3.2.1. Chitosan Scaffolds Synthesis 

For obtaining the chitosan scaffolds, each time 1.0 g of the biopolymer with 80% deacetylation degree was dissolved in an aquatic solution of acetic acid (4%) on a magnetic stirrer and was left until a homogenous mixture was obtained (1 h). In the next step, an appropriate amount of crosslinking agents and 10 mL of propylene glycol were added to each sample. Then, the reacting mixtures were placed in Prolabo Synthewave 402 microwave reactor. All syntheses were carried out in the field of microwave radiation, in various synthesis conditions (Table 1). Obtained chitosan hydrogels were washed out from the crosslinking agents’ residues, using distilled water. Finally, the hydrogels were frozen and lyophilized.

#### 3.2.2. Chemical Structure Analysis

Chemical structure of the products was evaluated by infrared spectroscopy. FT–IR/ATR analysis was performed using IR Thermo Nicolet Nexus X 470 spectrometer (diamond crystal ATR) (Thermo Fisher Scientific, Waltham, MA, USA). The range was between 400 and 4000 cm^–1^ with 32 scans and 4 cm^–1^ resolution.

#### 3.2.3. Porosity and Density Study

The density and porosity of the obtained chitosan materials were determined by isopropanol displacement because it did not wet the sample. The investigated biomaterials were placed into the previously measured volume of isopropanol. After a fixed time (5 min), the change in volume of the alcohol-impregnated aerogel was measured. Then, the studied chitosan scaffold was removed from the isopropanol. In the last step, the difference in isopropanol volume was measured. Based on this, the obtained data density (Equation (1)) and porosity (Equation (2)) was calculated using the following equations:

(1) $$d\;=\;\frac{W}{V_{2}-V_{3}}$$

(2) $$p\;=\;\frac{V_{1}-V_{3}}{V_{2}-V_{3}}\times 100\%$$

#### 3.2.4. Swelling Capability Study

To determine the swelling properties of the obtained biomaterials, samples were weighed and placed in distilled water. The samples were weighed after two time-periods (5 min and 24 h) and the swelling degree [%] was calculated according to Equation (3). Experiments were repeated 3 times.

(3) $$\%SD\;=\;\frac{W_{t}-W_{0}}{W_{0}}$$

where

%*SD*—swelling degree
*W_t_*—weight of the investigated sample after time = t, g
*W*_0_—initial weight of the investigated sample, g

#### 3.2.5. Scanning Electron Microscope (SEM) Analysis and X-ray Microanalysis

SEM analysis was performed using FEI QUANTA 650 FEG (ThermoFisher Scientific, Oregon, USA). Microphotographs were taken under pressure of 50 Pa and HV of 10.00 kV. X-ray microanalysis of the materials was performed using the energy dispersive spectroscopy method, using the FEI QUANTA 650 FEG microscope, equipped with an EDS detector (Thermo Fisher Scientific, Portland, OR, USA).

#### 3.2.6. Antioxidant Activity Study

Antioxidant properties of the prepared chitosan scaffolds were investigated by a standard DPPH method. For this purpose, a solution of DPPH in methanol was prepared so that the solution absorbance was 1.0 at 517 nm, using Aligent 8453 spectrophotometer. To determine the ability of free radical scavenging, 0.10 g of each sample was placed in 5 mL of DPPH solution and left in darkness for 1 h, with constant shaking. Then, the absorbance of each solution was measured at 517 nm. The percentage of the free radicals removed was calculated using the following Equation (4):

(4) $$\%S\;=\;\frac{A_{s}-A_{c}}{A_{c}}$$

where

%*S*—the % of the free radicals that were neutralized
*Ac*—the absorbance of the DPPH solution without the sample
*As*—the absorbance of the DPPH solution containing sample

#### 3.2.7. Cytotoxicity Study

VK2/E6E7 and HCT116 cells were grown on the tested scaffolds. Control cultures were grown under standard conditions. The cultures were grown on two types of scaffolds—one sterilized with ethylene oxide and one non-sterilized. Prior to plating the cells, each scaffold was rinsed for about 24 h in PBS, while the non-sterilized scaffold was rinsed in PBS with the addition of antibiotics. Next, the culture medium was added and placed in the incubator for about 24 h. The scaffolds were additionally coated with fibronectin for 24 h. About 200,000 cells were plated on scaffolds. The cultures were run on 6-well plates.

## 4. Conclusions

In this article, an attempt was made to obtain a novel chitosan-based scaffold dedicated to urogynecologic regenerative medicine applications. We successfully prepared the new crosslinked chitosan derivatives under microwave-assisted conditions. The crosslinking process was confirmed by the FT–IR method. The biomaterials exhibited excellent porosity and swelling abilities. They also had good antioxidant activity. However, surprisingly they appeared to be cytotoxic to both vaginal epithelial (VK2/E6E7) and HCT116 colorectal carcinoma cells. Such results suggest that the combination of crosslinking agents must be better adjusted and the purification process should be enhanced. In a future study, we will focus on the preparation of scaffolds crosslinked with the bifunctional acids of lower acidity, which would not negatively affect the cultured cells. Overall, it could be concluded that the choice of the right crosslinking agent was crucial for the chitosan biocompatibility maintenance and its effect on the chemical structure was superior to the porous morphology or swelling abilities. Development of the chitosan-based scaffolds applicable in pelvic organ prolapse (POP) treatment must be preceded by careful modifying agent characterization.

## Figures and Tables

**Table 1:** Chitosan scaffolds synthesis parameters.

Sample	Crosslinking Agent, g	High Boiling Solvent, mL	Chitosan, g	H_2_O, mL	Reaction Time, min
**1**	Adipic, 0.47 Malonic, 0.12	10	0.5	15	20
**2**	Adipic, 0.48 l-glutamic, 0.22	10	0.5	15	20
**3**	Adipic, 0.16 l-glutamic, 0.16 Malonic, 0.26	10	0.5	20	20
**4**	Adipic, 0.5 Levulinic, 0.5	10	0.5	20	20
