# Correction: Radwan-Pragłowska et al. The Potential of Novel Chitosan-Based Scaffolds in Pelvic Organ Prolapse (POP) Treatment through Tissue Engineering. *Molecules* 2020, *25*, 4280

**DOI:** 10.3390/molecules30163349

**Published:** 2025-08-12

**Authors:** Julia Radwan-Pragłowska, Klaudia Stangel-Wójcikiewicz, Marek Piątkowski, Łukasz Janus, Dalibor Matýsek, Marta Kot, Marcin Majka, Dalia Amrom

**Affiliations:** 1 Department of Biotechnology and Physical Chemistry, Faculty of Chemical Engineering and Technology, Cracow University of Technology, Warszawska 24 Street, 31-155 Cracow, Poland; marek.piatkowski@pk.edu.pl (M.P.); lukasz.janus@doktorant.pk.edu.pl (Ł.J.); 2 Gynecology and Oncology Department, Jagiellonian University Collegium Medicum, Kopernika 23, 31-501 Kraków, Poland; klaudia.stangel-wojcikiewicz@uj.edu.pl (K.S.-W.); daliamrom@gmail.com (D.A.); 3 Faculty of Mining and Geology, Technical University of Ostrava, 708 00 Ostrava, Czech Republic; dalibor.matysek@vsb.cz; 4 Transplantology Department, Jagiellonian University Collegium Medicum, Wielicka 265, 30-663 Kraków, Poland; marta.kot@uj.edu.pl (M.K.); mmajka@cm-uj.krakow.pl (M.M.)

## Addition of an Author

“Marta Kot” was not included as an author in the original publication [1]. Marta Kot conducted experiments to assess the cytotoxicity of scaffolds using various cell lines (VK2/E6E7, HCT116) and developed and interpreted the results and prepared the figures for the paper. The results of these experiments are included in Section 2.6 Cytotoxicity Study on page 7–10 in the published article.

The corrected Author Contributions statement appears here:

**Author Contributions:** Conceptualization, J.R.-P., K.S.-W., Ł.J., M.P. and M.M.; methodology, J.R.-P., M.P., M.M., Ł.J., M.K. and K.S.-W.; investigation, J.R.-P., Ł.J., M.P., M.M., M.K. and K.S.-W.; resources, M.P., Ł.J., M.M. and D.M.; writing—original draft preparation, J.R.-P., Ł.J. and D.A.; supervision, M.P., K.S.-W. and M.M. All authors have read and agreed to the published version of the manuscript.

Due to the expiration of Julia Radwan-Pragłowska’ previous email address, we have updated Julia Radwan-Pragłowska’ email to julia.radwan-praglowska@pk.edu.pl to facilitate reader contact.

The authors state that the scientific conclusions are unaffected. This correction was approved by the Academic Editor. The original publication has also been updated.

## References

[1] Radwan-Pragłowska J., Stangel-Wójcikiewicz K., Piątkowski M., Janus Ł., Matýsek D., Kot M., Majka M., Amrom D. (2020). The Potential of Novel Chitosan-Based Scaffolds in Pelvic Organ Prolapse (POP) Treatment through Tissue Engineering. Molecules.

